# Effects of Dosage, Comorbidities, and Food on Isoniazid Pharmacokinetics in Peruvian Tuberculosis Patients

**DOI:** 10.1128/AAC.03258-14

**Published:** 2014-12

**Authors:** Ana Requena-Méndez, Geraint Davies, David Waterhouse, Alison Ardrey, Oswaldo Jave, Sonia Llanet López-Romero, Stephen A. Ward, David A. J. Moore

**Affiliations:** a Laboratorio de Investigación de Enfermedades Infecciosas, Universidad Peruana Cayetano Heredia, Lima, Peru; b Barcelona Centre for International Health Research, CRESIB, Hospital Clinic-Universitat de Barcelona, Barcelona, Spain; c Department of Molecular and Clinical Pharmacology, University of Liverpool, Liverpool, United Kingdom; d Department of Molecular Parasitology, Liverpool School of Tropical Medicine, Liverpool, United Kingdom; e Servicio de Pneumología, Hospital Dos de Mayo, Lima, Peru; f TB Centre and Department of Clinical Research, Faculty of Infectious and Tropical Diseases, London School of Hygiene and Tropical Medicine, London, United Kingdom

## Abstract

Poor response to tuberculosis (TB) therapy might be attributable to subtherapeutic levels in drug-compliant patients. Pharmacokinetic (PK) parameters can be affected by several factors, such as comorbidities or the interaction of TB drugs with food. This study aimed to determine the PK of isoniazid (INH) in a Peruvian TB population under observed daily and twice-weekly (i.e., biweekly) therapy. Isoniazid levels were analyzed at 2 and 6 h after drug intake using liquid chromatography mass spectrometric methods. A total of 107 recruited patients had available PK data; of these 107 patients, 42.1% received biweekly isoniazid. The mean biweekly dose (12.8 mg/kg of body weight/day) was significantly lower than the nominal dose of 15 mg/kg/day (*P* < 0.001), and this effect was particularly marked in patients with concurrent diabetes and in males. The median maximum plasma concentration (*C*_max_) and area under the concentration-time curve from 0 to 6 h (AUC_0–6_) were 2.77 mg/liter and 9.71 mg·h/liter, respectively, for daily administration and 8.74 mg/liter and 37.8 mg·h/liter, respectively, for biweekly administration. There were no differences in the *C*_max_ with respect to gender, diabetes mellitus (DM) status, or HIV status. Food was weakly associated with lower levels of isoniazid during the continuation phase. Overall, 34% of patients during the intensive phase and 33.3% during the continuation phase did not reach the *C*_max_ reference value. However, low levels of INH were not associated with poorer clinical outcomes. In our population, INH exposure was affected by weight-adjusted dose and by food, but comorbidities did not indicate any effect on PK. We were unable to demonstrate a clear relationship between the *C*_max_ and treatment outcome in this data set. Twice-weekly weight-adjusted dosing of INH appears to be quite robust with respect to important potentially influential patient factors under program conditions.

## INTRODUCTION

Despite recent progress, global tuberculosis (TB) control is hampered by the impact of HIV coinfection (1, 2) and diabetes mellitus (DM) (3, 4) on the incidence of disease and treatment outcomes worldwide. Standardized short-course treatment regimens which are currently recommended by the WHO (5) remain the cornerstone of control strategies and may help to achieve high cure rates, although there is a need for proper evaluation of dosing schedules and of the optimal duration of treatment (6).

Several factors, including poor adherence (7), high bacillary burden (8), radiological cavitation (9), and DM (3), are associated with poor outcomes. HIV-positive people may be at risk of poorer outcomes during fully intermittent therapy without antiretroviral treatment (2) and remain at considerable risk of reinfection after successful treatment (10).

Debate continues about the impact of antituberculosis drug pharmacokinetics (PK) on clinical outcomes (11–15). Experimental and clinical studies have pointed to the influence of interpatient variability in key PK parameters, such as plasma area under the curve (AUC), on important outcomes, including treatment success and the emergence of resistance (16–19). These PK parameters may be affected by several factors, including age, gender, ethnicity, genetics, nutritional status, drug formulation and quality, and drug-drug interactions (20). Although data have accumulated on the impact of HIV on the PK of anti-TB drugs (11, 14), only a few studies have specifically examined the effect of DM, and their results have conflicted (21, 22).

Isoniazid (INH), one of the key agents in first-line TB therapy, has high early bactericidal activity (EBA) and exhibits prolonged postantibiotic effects (PAE) *in vitro*, which may be important for preventing the emergence of resistance during therapy (23). Its metabolism is controlled by the polymorphic *N*-acetyltransferase-2 (NAT2) locus, which results in relatively high interindividual variability in isoniazid PK (24). The maximum serum drug concentration (*C*_max_) of INH generally occurs between 1 and 2 h after drug intake, although food, particularly high-fat meals, may delay and reduce overall absorption.

Although INH is theoretically well suited to intermittent administration, there are few modern data on the PK of the drug during intermittent dosing (25, 26), particularly twice-weekly regimens, which are used in several Latin American countries. While this approach is effective (6) and may help to promote adherence, its PK robustness in the face of important patient factors, such as DM, HIV, acetylator phenotype, and food effect, has not been extensively studied. We report here the results of a field PK study in Lima, Peru, which aimed to evaluate plasma concentrations of isoniazid under daily and twice-weekly dosing in these important patient subgroups.

## MATERIALS AND METHODS

### Field methods.

This study was conducted in Lima, Peru, from July to December 2009. TB patients who were receiving directly observed first-line TB therapy through the national TB program and who had completed at least 15 days of treatment were invited to participate and provided written informed consent. Isoniazid in use in the Peruvian National Tuberculosis Program (PNTP) at the time of the study was sourced from IQ-Farma and the LCG corporation, both Peruvian companies. The PNTP recommends a daily dose of 5 mg/kg of body weight/day during the intensive phase of treatment and a biweekly dose of 15 mg/kg/day (maximum dose of 800 mg) during the continuation phase. Within the PNTP, TB drugs are not available as fixed-dose combinations. Patients with known HIV disease or DM were particularly sought, with the aim of recruiting at least 25 individuals for each subgroup. Blood sampling was performed 2 and 6 h after observed dosing under field conditions. In particular, intake of food and water was not controlled by the investigators. Patients were contacted again at the end of therapy and 6 months after treatment completion in order to evaluate the final treatment outcomes. This study was approved by the ethics committees of the London School of Hygiene and Tropical Medicine, Hospital Nacional Dos de Mayo, and Universidad Peruana Cayetano Heredia (UPCH) in Lima and the institutional review board of Dirección de Salud (DISA II) in Lima Sur (regional Ministry of Health).

### Laboratory methods.

Whole blood (10 ml) was drawn into lithium heparin tubes, kept in the dark, and transferred under refrigeration within 3 to 5 h to the research laboratory at UPCH. The samples were centrifuged at 2,000 rpm for 10 min, and plasma supernatant was removed and stored at −70°C prior to bioanalysis at the Liverpool School of Tropical Medicine. A total of 100 μl of plasma from each sample was assayed alongside a plasma calibration curve (range, 0 to 6,000 ng/ml) and quality control samples at low (40 ng/ml), medium (2,500 ng/ml), and high (5,000 ng/ml) concentrations of INH. Samples which tested above our highest level of quantification were diluted 1:4 and were reanalyzed with a fresh calibration curve and quality control samples.

Each sample underwent protein precipitation with 900 μl of an internal standard (IS) (acetonitrile containing 200 ng/ml of metformin). All the IS responses for the analytical runs carried out in our samples were <15% relative standard deviation, according to the bioanalytical guidelines approved by the FDA. The samples were vortexed for 20 s and were centrifuged at 14,000 × *g* for 20 min; 800 μl of supernatant was transferred to clean 5-ml soda-glass tubes and evaporated to dryness under nitrogen at 30°C. Dried-down samples were reconstituted in 100 μl of mobile phase (90% water, 10% methanol, and 0.3% formic acid) and were vortexed for 10 s. Reconstituted samples were transferred to insert vials and centrifuged at 4,700 × *g* for 5 min; 20 μl of the reconstitute underwent chromatographic separation on a Hypersil GOLD C_18_ column (150 by 4.6 mm, 3-μm particle size) (Thermo Scientific, Hemel Hempstead, United Kingdom) at 30°C using an isocratic gradient of 90% water, 10% methanol, and 0.3% formic acid at a rate of 300 μl/min. The high-performance liquid chromatography system was interfaced with a triple-quadrupole TSQ Quantum Access mass spectrometer (Thermo Scientific) with an atmospheric pressure chemical ionization (APCI) source. An E2M30 rotary vacuum pump (Edwards High Vacuum International, West Sussex, United Kingdom), an NM30LA nitrogen generator (Peak Scientific, Renfrewshire, United Kingdom), and 99% pure argon gas (10 liters) (BIP10; Air Products, Liverpool, United Kingdom) were used.

The mass spectrometer was operated in positive selective reaction monitoring (SRM) mode using transitions of *m*/*z* 138.2 to 121.1 for INH and 130.2 to 60.4 for IS, an optimized collision energy of 17 eV for INH and IS, a narrow scan width (0.1 *m*/*z*) and scan time (0.1 s) for all transitions, and the data collection system operating in centroid mode. The sheath and auxiliary gas flows (nitrogen gas) were 15 and 20 lb/in^2^, respectively. The capillary temperature within the ion source was maintained at 250°C, the discharge current was set to 5 μA, the spray voltage was set to 4.5 kV, and the collision pressure was 1.5 mTorr (argon). All standard curves were adequately described using an equal-weighted linear regression equation for INH using the data acquisition software LCquan version 2.5.6 (Thermo Scientific, Hemel Hempstead, United Kingdom). The correlation coefficient (*r*^2^) for all INH calibration curves exceeded 0.99. The lower limit of quantification (LLOQ) (10 ng/ml for INH) was accepted as the lowest point on the standard curve, with a signal-to-noise ratio of 5:1 and a coefficient of variation (CV) of <11% for INH; the CV ranged from 2% to 11% at all other calibration levels for INH. The determination of INH stability following three freeze-thaw cycles showed that all quality control samples were within a CV of 11% for INH concentrations.

### Data analysis.

For each patient, the *C*_max_ was defined as the higher of the two concentrations measured at 2 and 6 h, and the *T*_max_ was the time point at which the *C*_max_ occurred. PK parameters were obtained by noncompartmental analysis using the trapezoidal rule and the linear-up-log-down method. MIC data were not available, and no additional analysis of PK-pharmacodynamic (PD) parameters was developed. Although an internationally agreed-upon guideline for therapeutic drug monitoring is lacking, a normal INH *C*_max_ may be defined, by comparison with existing pharmacokinetic data, as 3 to 5 mg/liter after a 5-mg/kg daily dose and as 9 to 15 mg/liter after a biweekly dose of 15 mg/kg/day (20). A *C*_max_ level of <2 mg/liter after a 300-mg daily dose or a *C*_max_ level of <7 mg/liter after a 900-mg biweekly dose is regarded as inadequate and is an indication for dose adjustment, according to some experts (19). We categorized our PK data accordingly. For 5-mg/kg/day daily dosing, very low *C*_max_ levels were <2 mg/liter, low levels were 2 to 3 mg/liter, and normal levels were >3 mg/liter; for 15-mg/kg/day biweekly dosing, very low levels were <7 mg/liter, low levels were 7 to 9 mg/liter, and normal levels were >9 mg/liter. The chi-square test was used for the comparison of proportions, and the Student *t* test or Wilcoxon rank-sum test was used for continuous variables, depending on variable distribution. The data were analyzed with Stata (Stata Corp, College Station, Texas, USA) and R version 3.0.1 (R Foundation for Statistical Computing, Vienna, Austria).

## RESULTS

The general characteristics of the patients are described in Table 1. The PK data were not available for 6 out of 113 patients initially recruited due to inefficient processing of the sample or difficulties with venipuncture. In 4 of the remaining 107 patients, the 6-h sampling time was missed. Overall, 62 patients (57.9%) were sampled while receiving treatment in the intensive phase, and 45 were sampled in the continuation phase. As previously reported for this population (27), the patients with DM were significantly older and had a significantly higher body mass index (BMI) than the TB patients without DM (*P* < 0.001 for both).

**TABLE 1:** Participant characteristics by subgroup

Characteristic	TB (*n* = 52)^*a*^	DM-TB (*n* = 25)^*a*^	*P*	HIV-TB (*n* = 30)^*a*^	*P*	Total (*n* = 107)^*a*^
Sex (male)	26 (50)	16 (64)	0.2	26 (86.7)	**0.001**^*b*^	67 (63.81)
Age (yr)	29 (22.5–36)	50 (44–58)	**<0.001**	35 (30–38)	0.3	35 (26–44)
Confirmed TB^*c*^	40 (76.9)	21 (84)	0.5	17 (56.7)	0.055	78 (72.9)
BMI (kg/m^2^)^*d*^	22.9 (21.23–24.89)	27 (24.9–29.8)	**<0.001**	22 (20.8–24.6)	0.5	23.7 (21.5–25.8)
Chronic diarrhea^*e*^	0 (0)	0 (0)		2 (6.7)	0.059	2 (1.9)
Intestinal surgery^*f*^	4 (7.7)	4 (16)	0.3	2 (6.7)	0.9	10 (9.35)
Blood glucose level (mg/dl)	95^*g*^ (80–102)	119.5^*c*^ (111–211.5)	**<0.001**	92.5^*g*^ (81–102.5)	0.8	98^*c*^ (84–114)
Pathogenic parasites	4 (7.7)	1 (4)	0.5	4^*h*^ (14.3)	0.3	9^*e*^ (8.6)
Nonpathogenic parasites	16 (30.8)	12 (48)	0.1	8^*h*^ (28.6)	0.8	36^*e*^ (34.3)
Intensive phase treatment	33 (63.5)	13 (52)	0.2	17 (56.7)	0.5	62 (57.9)

a Values shown are the number of cases (% of total) or median (interquartile range). All the tests compare the DM-TB or HIV-TB groups with the TB-only (non-HIV non-DM) group. Continues variables were analyzed with the independent *t* test, and categorical variables were analyzed with the Pearson chi-square test.

b Bold type indicates a significant difference.

c Confirmed TB indicates microbiologically confirmed TB cases.

d BMI, body mass index.

e Chronic diarrhea was defined as persistence of liquid depositions for >15 days.

f Intestinal surgery was surgery related to a gastrointestinal tube.

g For two HIV patients, one DM patient, and two TB control patients, we were unable to test the blood glucose level.

h For two HIV patients, feces samples were not processed.

**FIG 1:**
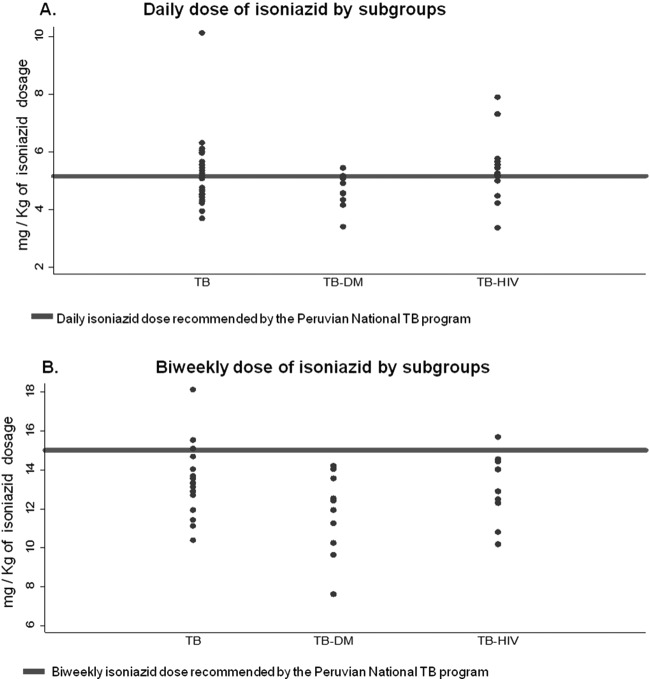
Daily (A) and biweekly (B) isoniazid doses, as recommended by the Peruvian National TB program.

The overall mean dose received during the intensive daily dosing phase was 5.15 mg/kg/day. Because of their higher BMI, the patients with DM received a lower daily dose of isoniazid than the patients with TB only (4.47 versus 5.3 mg/kg, respectively; *P* = 0.02, *t* test) (Fig. 1). During the continuation phase, the mean biweekly dose received on the treatment days was 12.8 mg/kg/day, which was significantly lower than the recommended dose of 15 mg/kg/day (*P* < 0.001); and 89% of the patients received doses lower than the nominal dosage. This effect was particularly marked in the TB-DM group (11.6 versus 13.3 mg/kg/day, respectively; *P* = 0.015) (Fig. 1). The mean maintenance phase dose of isoniazid received was lower in males than in females (12.22 versus 13.48 mg/kg/day, respectively; *P* = 0.008).

The *T*_max_ occurred at 2 h in 86.4% of intensive phase patients and in 90.7% of continuation phase patients. In the intensive phase patients, the median isoniazid concentration was 2.55 mg/liter (interquartile range [IQR], 1.67 to 3.5 mg/liter) at 2 h and was 0.59 mg/liter (IQR, 0.3 to 2.31 mg/liter) at 6 h. The median *C*_max_ was 2.77 mg/liter (IQR, 1.75 to 4.67 mg/liter). In continuation phase patients, the median concentrations of isoniazid at 2 and 6 h were 8.71 mg/liter (IQR, 4.01 to 23.27 mg/liter) and 3.1 mg/liter (IQR, 1 to 5.81 mg/liter), respectively. The median *C*_max_ was 8.74 mg/liter (IQR, 4.21 to 23.03 mg/liter). The percentage of patients in the intensive phase group that did not reach a reference value at 2 h after drug intake of at least 2 mg/liter (i.e., very low levels) was 34%; 33.3% of patients taking isoniazid biweekly did not achieve a reference value of 7 mg/liter at 2 h. The median AUC from 0 to 6 h (AUC_0–6_) was 9.71 mg·h/liter (5.87 to 13.31 mg·h/liter) for intensive phase patients and 37.8 mg·h/liter (19.2 to 82.26 mg·h/liter) for continuation phase patients. Since information about the NAT2 genotypes of participants was not available, a bivariate normal mixture model was fitted to the distribution of the half-lives to estimate the proportion of acetylator phenotypes from the data. Mixtures of normal distributions were fitted to PK parameter distributions using maximum likelihood in R. The best model for the distribution was bimodal, in which 53% of the population was estimated to be fast/intermediate acetylators with a mean half-life of 1.48 h, and 47% were estimated to be slow or intermediate acetylators with a mean half-life of 5.25 h.

**FIG 2:**
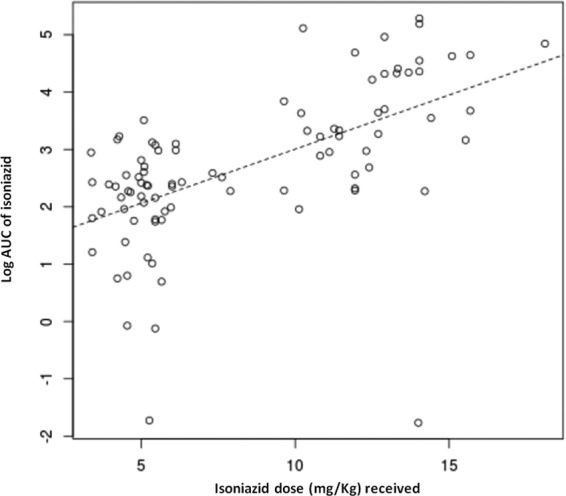
Relationship between dosage and exposure of isoniazid.

**FIG 3:**
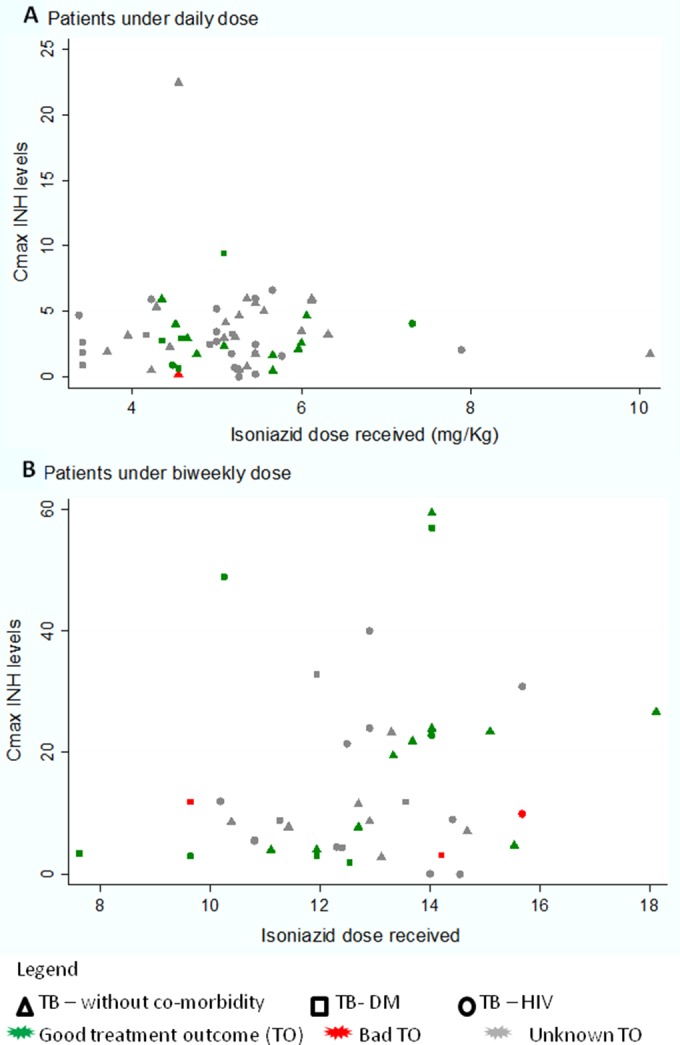
INH *C*_max_ levels by comorbidity and treatment outcome for patients who received a daily (A) or biweekly (B) dose.

The univariate analysis of factors influencing PK parameters is presented in Table 2. The relationship between the weight-adjusted dose and the PK exposure as measured by the AUC_0–6_ was demonstrated to be linear across the wide range of doses studied (Fig. 2). Information on food intake of the patients was available for only 48 patients, 12 of whom fasted for at least 2 h before taking the drugs, as recommended. Although the *C*_max_ and AUC_0–6_ appeared reduced in nonfasting patients, this was not a statistically significant finding (Table 2). Notably, none of the six patients in the intensive phase group who fasted before drug intake had 2-h concentration levels of <2 mg/liter, compared to 12/25 (48%) of intensive phase patients who did not fast (*P* = 0.04, Fisher' exact test), although this was not true in the maintenance phase group (33% fasting versus 22.2% nonfasting patients had 2-h concentration levels of <7 mg/liter; *P* = 0.4). In the univariate analysis, the *C*_max_ and AUC_0–6_ were unaffected by age, gender, DM, or HIV in either the intensive or the continuation phase (Table 2; Fig. 3). Multivariate models with log-transformed PK parameters as dependent variables were used to further evaluate the effects of the comorbidities of interest, while taking into account the effect of the dose. For the AUC_0–6_ and *C*_max_, the isoniazid dose received was the most significant covariate (*P* = 0.028 and *P* = 0.029, respectively), whereas comorbidity (i.e., having HIV infection or DM), age, sex, fasting, and intestinal parasitosis did not significantly influence the PK parameters (data not shown).

**TABLE 2:** Determinants of the INH *C*_max_ and AUC_0–6_ in daily and twice-weekly doses

Determinant	Daily dose of INH						Twice-weekly dose of INH					
	*n*	Median *C*_max_ (mg/liter)	*P* value	*n*^*b*^	AUC_0–6_ (mg·h/liter)	*P* value	*n*	Median *C*_max_ (mg/liter)	*P* value	*n*^*b*^	AUC_0–6_ (mg·h/liter)	*P* value
TB comorbidity^*a*^												
DM	11	2.6	1	11	10.39	0.6	13	4.4	0.3	11	54.26	0.2
HIV	16	2.6	0.8	13	9.72	0.6	13	9.9	1	12	53.64	0.6
TB alone	32	3		27	11.05		18	8.6		16	62.45	
Sex												
Male	36	2.8	1	31	10.23	0.9	29	8.6	0.2	26	46.28	0.2
Female	23	2.8		20	11.1		15	21.4		13	79.71	
Fasting status with therapy												
Fasting	6	3.1	0.4	6	11.06	0.2	6	16.6	1	4	71.52	1
Nonfasting	25	2.1		20	6.96		9	7.7		8	53.17	
Age group												
18–30 yrs	22	3	0.8	20	10.61	0.6	17	9.9	0.9	15	40.51	0.7
31–40 yrs	23	2.5		18	8.77		12	7.4		10	31.45	
>40 yrs	14	2.9		13	9.71		15	8.8		14	28.32	
Intestinal parasites												
Yes	4	2.1	0.6	3	4	0.3	5	9	0.9	5	34.81	0.9
No	53	3		46	10.12		39	8.6		34	38.65	
Bacteriologically confirmed TB												
Yes	44	2.8	0.6	36	9.6	1	31	9.9	0.9	26	38.9	0.6
No	15	2.5		15	9.7		13	8.6		13	19.6	
Overall median	59	2.8		51	9.71		44	8.7		39	37.81	

a The TB comorbidity group compared the DM-TB or HIV-TB group with the TB-alone (non-HIV non-DM) group. All the variables were analyzed with the nonparametric Wilcoxon test, and the Kruskal-Wallis test was used for the age group variable.

b The numbers related to the *C*_max_ are slightly different than the numbers related to the AUC, because we were unable to calculate the AUC in cases of undetectable levels or delayed absorption (according to the *C*_max_ at 6 h).

The MIC data were unavailable; therefore, the PK-PD parameters were not estimated.

Treatment outcomes were available 6 months after the completion of therapy in 99 of 107 patients. After the exclusion of 10 patients diagnosed with multidrug-resistant TB, 26 who either abandoned treatment or did not properly complete treatment, and 22 others who lacked a microbiologically confirmed diagnosis, 41 patients were included in the treatment outcome subanalysis. A total of 37 patients were regarded as cured, and 4 had an unfavorable outcome; two patients (one who was positive for HIV) died during treatment, one patient had a drug-susceptible relapse within 6 months after an apparent cure, and one patient experienced prolonged treatment for persistent TB despite treatment.

In samples of both intensive and maintenance phase groups, there was no significant difference in the *C*_max_ of patients with a good treatment outcome compared to patients who had treatment failure or relapse. Three of the four patients who had unfavorable outcomes had very low *C*_max_ levels when sampled in either the intensive or continuation phase compared to 14/34 (41.2%) of those with favorable outcomes who had very low levels.

## DISCUSSION

We aimed to study the INH PK under real-life conditions in the PNTP using sparse PK sampling during daily and twice-weekly dosing. The range of INH doses included in the study was wider than in most published PK studies and clearly demonstrates the linearity of INH PK with weight-adjusted doses.

With the two dose regimens, the INH PK was highly variable, with >30% of patients failing to exceed a *C*_max_ deemed very low compared with those in other PK studies. A major finding was that most patients during the continuation phase received a weight-adjusted biweekly dose of INH below the PNTP-recommended 15-mg/kg/day dose. This was a particular problem in TB patients with DM who had a higher BMI, but we were unable to determine whether the lack of dose adjustments to account for weight gain during treatment may also have contributed. These observations have since led the PNTP to change the maximum biweekly isoniazid dose in the continuation phase from 800 mg to 900 mg and to reemphasize the importance of weight-based dosing.

We examined several other factors that might also explain the high interindividual variability we observed. Data on fasting prior to the administration of drugs were limited to a subset of patients, but in this underpowered subanalysis, which was not statistically significant, the observed PK parameters were lower for those who did not fast. Neither DM nor HIV independently influenced the INH PK. The DM population in the twice-weekly dosing group showed lower INH exposure, but this was explained by the higher BMIs. The sparse sampling scheme limited our ability to detect changes in the rate of absorption reflected in the *T*_max_ or *C*_max_. If such altered absorption exists, it did not seem to influence the overall AUC_0–6_, notwithstanding the reduced precision of calculating an AUC using two time points versus more enriched time point sampling. We did not obtain NAT2 genotype or metabolite data to accurately classify individuals by acetylator status. However, the population distribution of isoniazid half-lives was clearly bimodal. Even though the underlying distribution of acetylator phenotype is, in fact, trimodal (24), our data did not support a clear differentiation between intermediate and slow acetylators. More than half of the patients were predicted to be fast acetylators, which may have also contributed to low and variable INH exposure. Little information is currently available on acetylator status in Latin America, especially in Peru, where the *4 allele associated with the fast acetylator phenotype, and which is overrepresented in Asia, may be common (28). Future studies should address this issue with genotyping or metabolite data.

Efficacy targets for PK parameters for anti-TB drugs have historically been based on reference therapeutic ranges derived from achieved PK alone (20). The relevance of these to short- or long-term outcomes remains unclear. There were several studies in the 1960s that related the treatment outcome to PK and particularly to INH concentrations (29–31). Gangadharam et al. showed that the therapeutic efficacy increased above a critical peak concentration of “about 3 μg/ml” of INH (31). Another study supporting these data showed that early bactericidal activity (EBA) leveled off at a 2-h INH concentration between 2 and 3 μg/ml (32). Weiner et al. also reported low INH concentrations associated with poorer treatment outcome in patients who received once-weekly INH with rifapentine but not in those who received twice-weekly INH with rifapentine (26), which might serve as a surrogate for what should be expected if there was any noncompliance during the daily therapy. On the other hand, most studies of longer-term outcomes have not reported associations with INH PK parameters; instead, pyrazinamide or rifampin is usually implicated (10, 11, 14, 15, 17, 18). In this study, cutoffs suggested in the literature were not associated with poor outcomes. However, the relationship of the INH AUC with drug effect (at least as measured by early bactericidal activity) is well defined (28), which suggests that the variability in PK that we observed might be of clinical significance, especially during intermittent dosing.

Plasma exposure as measured by the median AUC_0–6_ was comparable with the median AUC_0–12_ described for patients with favorable treatment outcomes in two Tuberculosis Trials Consortium (TBTC) studies (28 mg·h/liter in “study 22” [26] and 48.8 mg·h/liter in “study 23” [25]), which used the same biweekly dosing regimen. However, our study lacked the power to detect small changes in important outcomes, such as the emergence of resistance and treatment failure/relapse. Moreover, since we were unable to measure the MICs, which is key for calculating the PK-PD parameters of the bactericidal activity for isoniazid (17), we were unable to evaluate the effect upon clinical outcome.

Conversely, one should also take into account that the combination therapy (with rifampin, ethambutol, or pyrazinamide) might have positively influenced the treatment outcome. Although this synergistic effect can potentially be measured through a microdilution checkerboard assay (33), drug susceptibility testing is done individually for each drug (34). Thus, any effect of a low INH concentration might be overcome by the effect of the other agents and thus not directly influence treatment outcome.

The rationale for the intermittent administration of drugs in the continuation phase is based on *in vitro* studies, which have suggested that antituberculosis drugs, particularly INH, demonstrate a significant and prolonged postantibiotic effect which permits an extended dosing interval (23). While the role of intermittent regimens in the intensive phase of treatment was recently questioned (35), no important differences in treatment outcome were noted in two meta-analyses of clinical trials between daily and intermittent dosing during the continuation phase, whether administration was twice or thrice weekly (6, 36). While updated WHO treatment guidelines no longer promote twice-weekly dosing, this preference appears largely based on consideration of the redundancy of the number of doses in the regimen rather than on the results of PK-PD analysis (5). Our data suggest that weight-based twice-weekly dosing remains a pharmacologically appropriate strategy in this context, and this dosing strategy appears to be quite robust with respect to the important potentially influential patient factors studied here, at least in the PNTP.

We acknowledge that our study had a number of limitations. For logistical reasons, we limited sampling to two time points (2 and 6 h), which facilitated the real-life nature of the work but limited the precision and accuracy of the *C*_max_, *T*_max_, and AUC_0–6_ estimates. We were also unable to adequately control or reliably measure the concomitant food intake of all the patients. The study lacked power to assess the full impact of PK parameters on long-term outcomes for several reasons. First, in our data set, TB diagnosis was not confirmed in all cases, particularly in HIV-TB patients, and this restricted our clinical outcome analysis to a smaller subgroup than intended (41 patients), which reduced the power of the study to detect such an effect had it been present. Second, no intermediate bacteriological data or susceptibility data for the pathogen, such as the MIC, were available, which should be taken into account to predict clinical efficacy. Larger studies using more sophisticated sparse-sampling strategies would be needed to better understand the relationship between INH PK and long-term outcomes. In this real-life PK study, variability in INH exposure was driven largely by weight-adjusted dose, a relatively high proportion of fast acetylators, and a possible food effect, whereas important comorbidities, such as DM and HIV, had no demonstrable additional impact on INH PK. Although the *C*_max_ was low in up to 30% of the participants, the AUC_0–6_ values were comparable with those of other studies and were not associated with poor outcomes. In particular, despite high interindividual variability in exposure, twice-weekly weight-adjusted dosing of INH appears quite robust with respect to important patient factors under program conditions.
